# Clinical and Biologic Correlates of ADORA2A Transcriptomic Expression in Cancer

**DOI:** 10.3390/ijms25094742

**Published:** 2024-04-26

**Authors:** Aditya Shreenivas, Daisuke Nishizaki, Suzanna Lee, Sarabjot Pabla, Mary Nesline, Jeffrey M. Conroy, Paul DePietro, Shumei Kato, Razelle Kurzrock

**Affiliations:** 1 Department of Oncology, Medical College of Wisconsin Cancer Center, Milwaukee, WI 53226, USA; 2 Center for Personalized Cancer Therapy and Division of Hematology and Oncology, Department of Medicine, UC San Diego Moores Cancer Center, La Jolla, CA 92037, USA; 3 Labcorp Oncology, Buffalo, NY 14203, USA; 4 WIN Consortium, 24, rue Albert Thuret, 94550 Chevilly-Larue, France; 5 Department of Medical Oncology, University of Nebraska, Omaha, NE 68105, USA

**Keywords:** ADORA2A, immune checkpoints, adenosine pathway

## Abstract

ADORA2A (adenosine A2a receptor) and ADORA2B propagate immunoregulatory signals, including restricting both innate and adaptive immunity, though recent data also suggest a tumor suppressor effect in certain settings. We evaluated the RNA expression from 514 tumors in a clinical-grade laboratory; 489 patients with advanced/metastatic disease had clinical outcome correlates. Transcript expression was standardized to internal housekeeping genes and ranked (0–100 scale) relative to 735 specimens from 35 different cancer types. Transcript abundance rank values were defined as “low/moderate” (0–74) or “high” (75–100) percentile RNA expression ranks. Overall, 20.8% of tumors had high ADORA2A (≥75 percentile RNA rank). The greatest proportion of high ADORA2A expressors was found in neuroendocrine and breast cancers and sarcomas, whereas the lowest was found in colorectal and ovarian cancers, albeit with patient-to-patient variability. In multivariable logistic regression analysis, there was a significant positive correlation between high ADORA2A RNA expression and a high expression of the immune checkpoint-related molecules PD-1 (*p* = 0.015), VISTA (*p* ≤ 0.001), CD38 (*p* = 0.031), and CD39 (*p* ≤ 0.001). In 217 immunotherapy-treated patients, high ADORA2A did not correlate significantly with progression-free (*p* = 0.51) or overall survival (OS) (*p* = 0.09) from the initiation of the checkpoint blockade. However, high versus not-high ADORA2A transcript expression correlated with longer OS from the time of advanced/metastatic disease (N = 489 patients; (HR 0.69 (95% CI 0.51–0.95) (*p* = 0.02)). Therefore, high ADORA2A transcript levels may be a favorable prognostic factor, unrelated to immunotherapy. Importantly, ascertaining co-expression patterns of ADORA2A with PD-1 and VISTA in individual tumors as a basis for the precision co-targeting of ADORA2A and these other checkpoint-related molecules warrants investigation in clinical trials.

## 1. Introduction

The adenosine A2a receptor (ADORA2A) is a member of the guanine nucleotide-binding protein (G protein)-coupled receptor (GPCR) superfamily [1]. ADORA2A and ADORA2B are protein coding genes of A2A or A2B adenosine receptors, respectively. ADORA2A is present at the q11.23 location on chromosome 22 [2]. These receptors use adenosine as the preferred endogenous agonist and interact with G proteins to increase intracellular cAMP levels. They play a vital role in protein biological functions, including, but not limited to, cardiac rhythm function, cerebral and kidney circulation, pain regulation, and sleep, and have been implicated in neurodegenerative and inflammatory disorders.

Of special interest, the conversion of extracellular adenosine triphosphate (ATP) into extracellular adenosine is a form of an immune checkpoint [3]. This process interferes with anti-tumor immune responses by preventing the pro-inflammatory action of ATP and by engaging adenosine signaling in immune cells and endothelial cells. Blay et al. reported that the concentration of adenosine is significantly increased compared to adjacent tissues in the tumor microenvironment (TME), creating an immune shield that helps the tumor fight off attacks from the immune system [4]. Targeted blockages of one of the main effectors of this pathway, the adenosine receptors responsible for elevating cAMP levels, can promote anti-tumor immunity and can lead to the enhanced efficacy of immune checkpoint inhibitors [3,5].

There are four subtypes of adenosine receptors (A1, A2A, A2B, and A3), which belong to the family of G protein-coupled receptors (GPCR), and all of them can be activated by extracellular adenosine. A1, A2A, and A2B receptors are highly homologous among species, whereas A3 receptors vary substantially. In terms of the affinity of ligand binding, A1, A2A, A2B, and A3 receptors have high affinity, whereas A2B shows a low binding affinity for adenosine [6]. Mittal et al. demonstrated that higher ADORA2B gene expression levels correlate with poorer overall survival in a triple-negative breast cancer subset, but not in luminal A, B, or HER2+ subsets [7].

ADORA2A plays an important regulatory role in the adaptive immune system. Just like programmed cell death-1 (PD-1) and cytotoxic T-lymphocyte-associated protein-4 (CTLA-4) receptors, it suppresses the immunologic response and prevents associated tissue damage. Ohta and colleagues were amongst the first to show that the blockade of the adenosine-A2AR-cyclic AMP axis can enhance T cell-mediated tumor regression in multiple in vivo models of cancer [8]. Multiple other studies have shown that A2A is a promising target for novel immunotherapies, and the direct/indirect inhibition of these receptors can lead to a sustained immunological tumor response. Investigators are also exploring the potential of combination therapies using A2A receptor antagonists and other checkpoint inhibitors such as anti-PD-1/PD-L1-based therapies, since adenosine receptor 2A blockade can enhance the effectiveness of anti-PD-1/PD-L1-based therapies by enhancing the anti-tumor T cell response [9,10]. Adenosine also plays an important role in changing the tumor microenvironment; it is present at low concentration levels in the interstitial fluids of unstressed tissues, but levels can rapidly increase in response to pathophysiological conditions such as hypoxia, ischemia, inflammation, or tissue injury. Increased adenosine can lead to immunosuppression within the tumor microenvironment [3,4,11].

**Figure 1:**
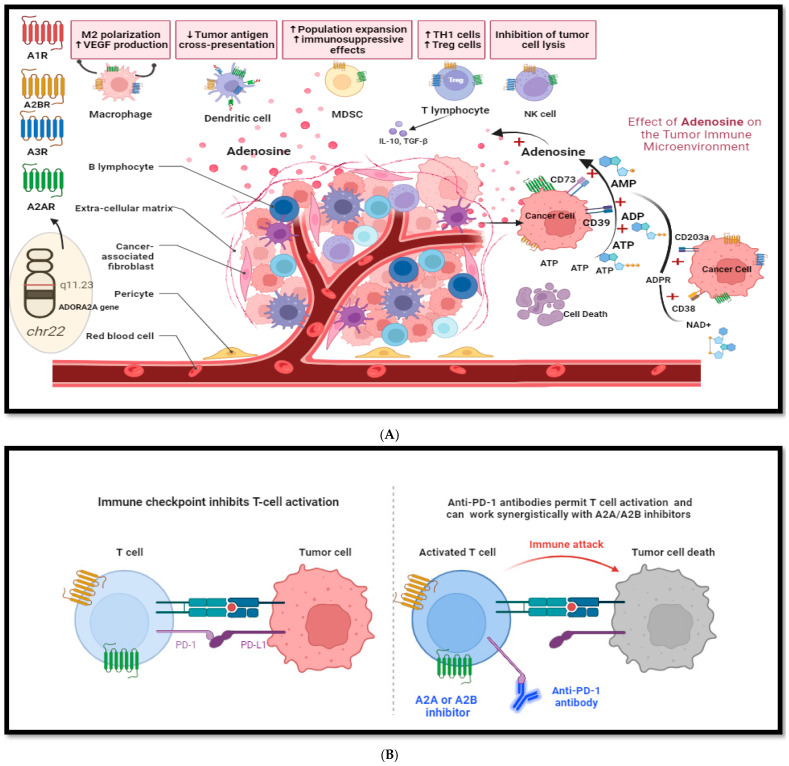
Impact of adenosine on the tumor-immune microenvironment (A2AR = ADORA2A) (created on Biorender.com, accessed on 20 April 2024). Panel (**A**) legend: adenosine acts as a critical immunosuppressive factor that accumulates in the tumor microenvironment. It is present at low nanomolar levels in the interstitial fluids of unstressed tissues, but its level can rapidly increase in response to pathophysiological stress factors like hypoxia, ischemia, inflammation, or tissue injury. Stress factors and cell death lead to extracellular aggregation of adenosine triphosphate (ATP) in the tumor microenvironment. Figure inside the bubble depicts the location of ADORA2A gene located at q11.23 in chromosome 22, which codes for A2A receptor. Some ectonucleotidases expressed on the cell surface of tumor and immune cells, such as CD39 and CD73, catalyze the conversion of extracellular ATP to adenosine. In the canonical pathway, ATP accumulating in the extracellular domain is converted to ADP and AMP by CD39 and, subsequently, the hydrolysis of AMP to adenosine by CD73. In the non-canonical pathway, NAD+ substrate is converted to ADP-ribose (ADPR) via CD38, which is then converted to AMP by CD203a and then hydrolyzed by CD73 to produce adenosine. Adenosine release increases or decreases (depicted by arrows) the effect of immune cells in tumor microenviroment. High levels of adenosine lead to immunosuppression by decreased tumor antigen cross presentation by dendritic cells, increased immunosuppressive effects of myeloid derived suppressor cells, increased TH1, T regulatory cells, and the inhibition of tumor cell lysis by natural killer (NK) cells. It also leads to increased VEGF production by macrophages and angiogenesis. Panel (**B**) legend: conceptualization of possible strategies to mitigate adenosine pathway-mediated immune resistance by combining immune checkpoint and A2A/A2B adenosine receptor inhibitors (A2A receptor = ADORA2A).

Some ectonucleotidases such as CD39 (also known as ecto-nucleoside triphosphate diphosphohydrolase 1, E-NTPDase1) and CD73 (also known as ecto-5′-nucleotidase, Ecto5′NTase), which are expressed on the cell surface, catalyze the conversion of ATP to AMP (adenosine monophosphate) and of AMP to adenosine, respectively (Figure 1, panel A [2,12]). While AMP production is thought to be predominantly mediated by CD39, an alternative source of AMP in this cycle is the conversion of NAD+ by CD38 and CD203a receptors [12,13]. The conversion of ATP to AMP to adenosine can be successfully targeted by A2A adenosine receptor inhibition [14]. CD73 is expressed on regulatory T (Treg) cells and various stromal cells in the bone marrow, such as mesenchymal stem cells, fibroblasts, and endothelial cells, whereas CD39 is expressed by regulatory T and B cells. Adenosine concentrations in the TME can increase multiple folds in response to stressors such as hypoxia, leading to immunosuppression by tumor tissues [15]. CD73 is essential in increasing adenosine production, which promotes cancer growth and metastasis by activating the PI3K/AKT signaling pathway inside the tumor cell [16]. Extracellular adenosine binds to A2AR and further activates Rap1, which then recruits P110β to the plasma membrane, triggers the production of PIP3, and leads to AKT phosphorylation. This process ultimately leads to angiogenesis, anti-apoptosis, and epithelial–mesenchymal transition [16]. In mouse models, the co-inhibition of CD73 and A2A receptor signaling can improve anti-tumor immune responses [17]. Adenosine signaling is also active inside immune cells. The A2A receptor is a dominant adenosine receptor, so its inhibition can downregulate the immunosuppressive effect of adenosine [18]. Blocking A2A adenosine receptors can also lead to enhanced interferon-gamma levels, the maturation of NK cells, and a cytotoxic CD8+ T cell response, further leading to tumor suppression [12]. However, paradoxically, ADORA2A may also have tumor-suppressive functions in certain settings [19].

The rationale of conducting this study was to utilize transcriptomics to study ADORA2A expression in various malignancies and its correlation with other immunomodulatory molecules, as well as with clinical outcomes.

We hypothesize that ADORA2A transcriptomic expression will significantly vary across different tumor types, and that a high expression of ADORA2A will have a correlation with a high expression of certain other immune checkpoint-related molecules.

## 2. Results

**Patient characteristics:** In total, 514 tumor samples were evaluated, including 489 with extensive clinical annotation focused on the advanced/metastatic setting. The median age of patients in the dataset was 61 years. The most frequent tumor types assessed were colorectal cancer (N = 140), breast cancer (N = 49), ovarian cancer (N = 43), and pancreas cancer (N = 55). Additional details are provided in Supplementary Table S1.

**Figure 2:**
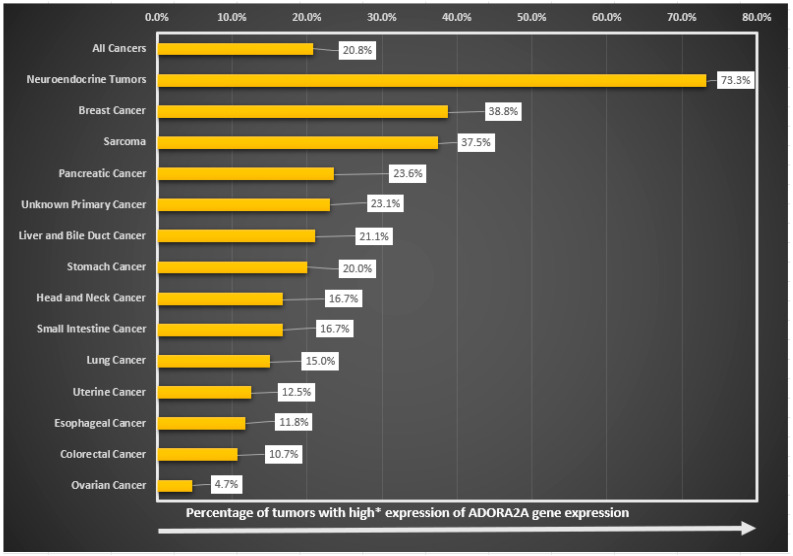
High RNA expression of ADORA2A genes across tumor subtypes (N = 514 patients). Percent shown reflects percent of tumors with high ADORA2A mRNA gene expression. Tumor subtypes with less than 10 samples were not included in this graph. * High expression refers to ≥75th percentile rank.

**The highest proportion of high ADORA2A RNA expressors was found in neuroendocrine, breast, and sarcoma tumors.** Of 514 tumors, 105 (20.8%) tumors had high ADORA2A (≥75 percentile RNA rank). Cancers with the highest proportion of ADORA2A transcriptomic expression were neuroendocrine (73%; 11/15 patients), breast (38.8%; 19/49), sarcoma (37.5%; 9/24), pancreatic (23.6%; 13/55), and carcinoma of unknown primary (23.1%, 3/13) (Figure 2). The correlation between high ADORA2A and neuroendocrine, breast, and sarcomas remained significant in multivariate analysis ((Table 1): neuroendocrine cancer, odds ratio (95% CI), 19.3 (5.53–80.4), (*p* < 0.001); breast cancer, odds ratio (95% CI), 4.43 (2.01–9.78), (*p* < 0.001); and sarcoma, odds ratio (95% CI), 3.34 (1.21–8.91), (*p* = 0.017)).

**High ADORA2A RNA expression negatively correlated with colorectal and ovarian cancer.** Only 10.7% and 4.7% of colorectal and ovarian cancers, respectively, expressed high ADORA2A levels (≥75 percentile RNA rank) (Table 1 and Figure 2). This negative correlation was significant in multivariate analysis ((Table 1): colon cancer, odds ratio (95% CI), 0.49 (0.24–0.96), (*p* = 0.044); and ovarian cancer, odds ratio (95% CI), 0.17 (0.02–0.67), (*p* = 0.028)).

**ADORA2A RNA expression showed individual variability between and within tumor types.** We found a variability of ADORA2A expression within tumor types. For instance, while 73.3% of neuroendocrine tumors expressed high ADORA2A, 26.7% expressed low/moderate ADORA2A. Similarly, while 37.5% of sarcomas expressed high ADORA2A, 62.5% expressed low/moderate ADORA2A. This pattern, reflecting individual variability, was seen in all cancer types analyzed (Figure 2).

**High ADORA2A RNA expression correlated significantly and independently with a high expression of PD-1, VISTA, CD38, and CD39.** We analyzed the relationship between ADORA2A and several immune checkpoint-related molecules (PD-L1, PD-1, PD-L2, CTLA-4, LAG3, VISTA, TIM-3, IDO1), as well as CD38 and CD39, the latter two because of their ability to generate immunosuppressive metabolites such as adenosine. High ADORA2A RNA expression correlated with a high RNA expression of PD-L1, PD-1, PD-L2, CTLA-4, LAG-3, VISTA, TIM-3, IDO1, CD38, and CD39 (Table 1). A statistically significant association was not observed between ADORA2A RNA expression and other immune biomarkers such as TMB ≥10 mutations/mb, PD-L1 IHC (CPS score ≥ 1), and MSI.

Multivariate analysis was then performed on variables with *p*-values ≤ 0.05 in univariate analysis to ascertain features independently correlated with high ADORA2A expression. Among the variables selected in univariate analysis, PD-1 (odds ratio (95% CI), 2.55 (1.19–5.45), (*p* = 0.015)), VISTA (odds ratio (95% CI), 3.05 (1.75–5.36), (*p ≤* 0.001)), CD38 (odds ratio (95% CI), 2.24 (1.07–4.63), (*p* = 0.031)), and CD39 (odds ratio (95% CI), 3.54 (1.93–6.54, (*p* ≤ 0.001)) remained positively associated with ADORA2A expression in multivariate analysis.

**Figure 3:**
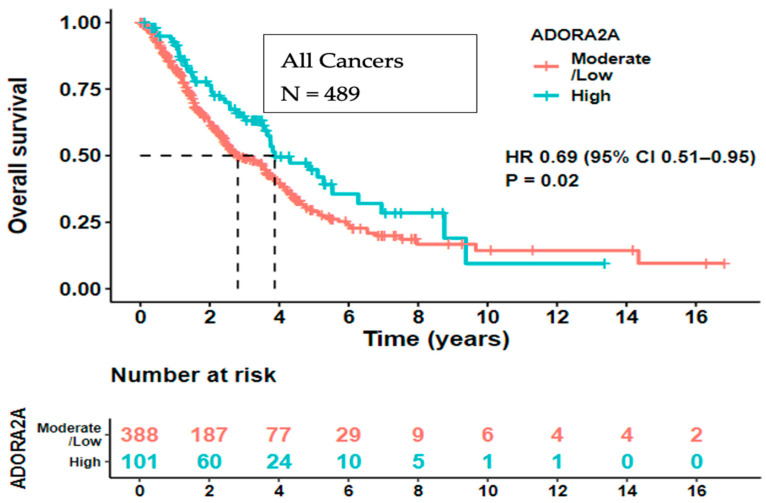
Kaplan–Meier survival curves of all cancer patients and patients that never received immunotherapy in the database.

**High ADORA2A RNA expression was a prognostic factor for longer OS from the time of advanced/metastatic disease.** Kaplan–Meier curves were also plotted to analyze the survival data of 489 tumor samples (25 of 514 patients were not included in the analysis due to missing clinical data). Patients with high ADORA2A transcript expression in their tumors had longer OS (from the time of advanced/metastatic disease) compared to those with low/moderate ADORA2A expression (HR 0.69 (95% CI, 0.51–0.95), (*p* = 0.02)) (Figure 3).

For all cancer patients that had clinical data (N = 489), OS was calculated from the date of advanced stage/metastatic disease to the date of last follow-up or death, stratified by ADORA2A levels. High refers to an RNA/transcript expression level ≥ 75th percentile of controls. Low/moderate refers to a transcript/RNA expression level < 75th percentile of controls. Patients with high ADORA2A RNA expression had significantly longer OS compared to moderate/low expression (*p* = 0.02).

**Figure 4:**
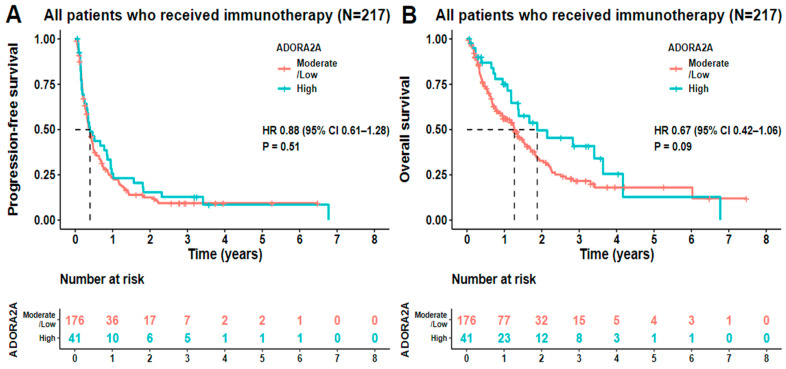
Kaplan–Meier curves for PFS and OS of patients treated with immunotherapy. (**A**,**B**) For patients that were treated (N = 217) with immunotherapy, PFS was defined from the date of treatment initiation to the date of the earliest of progression or death from any cause, stratified by ADORA2A. OS was calculated from date of treatment initiation to the date of last follow-up or death, stratified by ADORA2A levels. High refers to RNA/transcript expression level ≥ 75th percentile of controls. Low/moderate refers to transcript/RNA expression level < 75th percentile of controls. Patients with high ADORA2A RNA expression showed no significant difference in PFS (*p* = 0.51) or OS (*p* = 0.09) compared to moderate/low expression after immune checkpoint blockade immunotherapy.

**ADORA2A transcript levels did not predict the outcome of immune checkpoint therapy**. We also evaluated the impact of immune checkpoint inhibitor-based therapies (mainly anti-PD-1/PD-L1) on tumors with high ADORA2A RNA gene expression and plotted predictive Kaplan–Meier curves from the date of treatment initiation to the date of last follow-up or death. Notably, we found no statistically significant difference in PFS (*p* = 0.51) or OS (*p* = 0.09) between high and low/moderate ADORA2A RNA gene expressors in the 217 immunotherapy-treated individuals (Figure 4A,B).

## 3. Discussion

Immune checkpoint inhibitors such as PD-1/PDL-1 and CTLA-4 blockers have brought about a paradigm shift in the management of advanced cancers. Despite these noteworthy developments, cancers continue to manifest primary or secondary resistance to treatment with immune checkpoint blockers; hence, novel therapeutic approaches are required to manage these cases. Mitigating critical immune escape mechanisms such as the increased production of adenosine can open the doors to the development of multiple combination immunotherapeutic strategies.

Importantly, although certain tumor types such as neuroendocrine and breast cancers, as well as sarcomas, were over-represented amongst tumors with high ADORA2A expression in our study, and colorectal and ovarian cancers were underrepresented amongst tumor types with high ADORA2A, transcript levels varied from tumor to tumor, both between and within histologies. This observation indicates that, although certain patterns could be observed, an accurate ascertainment of tumor expression levels requires individual testing.

PD-L1 expression assessed through IHC is a well-known (albeit imperfect) predictive marker for immunotherapy [20,21]. There is also evolving evidence that PD-1-expressing tumor-infiltrating lymphocytes (TILs) and high PD-1 messenger RNA expression could be important predictors of PD-1/PD-L1 antibody responsiveness [22,23]. In our study, high ADORA2A expression was independently correlated with high PD-1 transcript expression, suggesting that mitigating the checkpoint effect in some cancers might require a combination of adenosine pathway inhibitors together with anti-PD-1 antibodies (Figure 1, panel B). Supporting this notion is prior data suggesting that an increased expression of adenosine 2A receptors in metastatic renal cell carcinoma is associated with a poorer response to anti-PD-1/anti-CTLA-4 antibodies [24]. Moreover, an A2AR antagonist for cancer treatment demonstrated clinical anti-tumor activity when given with an anti-PD-L1 as combination therapy in patients with refractory renal cell cancer. Of interest, responding tumors possessed an adenosine-regulated gene expression signature in pretreatment tumor biopsies [25,26]. Even so, in our study, high ADORA2A expression was not correlated with immunotherapy outcomes. One important difference between our study and the prior study was that the prior report focused on renal cell carcinoma, while we examined multiple tumor types.

As expected, there was a clear (significant and independent) correlation between high ADORA2A RNA expression and CD38 and CD39 expression, both of which play an essential role in the adenosine pathway. This is an important finding, as ongoing clinical trials are exploring combining novel agents that target CD38/CD39 with A2A or A2B adenosine receptor inhibitors to suppress adenosine production and improve tumor response (Supplementary Table S2) [27]. Over the years, in addition to PDL1/PD-1 and CTLA-4, a wealth of novel immune checkpoint targets has emerged. The V-domain Ig suppressor of T cell activation (VISTA) is one such target, which is expressed on resting CD4+ T cells and myeloid cells. It acts as a coinhibitory receptor and can negatively regulate T cell activation. High ADORA2A RNA expression positively correlated with VISTA in our analysis [28]. To our knowledge, there are no studies to explain the mechanism of this association. However, earlier studies from our group have also shown that a high RNA expression of VISTA could be a marker for resistance to anti-PD1/PDL1-based therapies [28,29,30]. Figure 1A,B illustrates the interaction between adenosinergic and immune pathways, which encompasses ectonucleotidases such as CD38/39 and immune checkpoints like PD1 and ADORA2A. In the canonical pathway, CD39 converts extracellular ATP to adenosine, which suppresses T cell receptor functions via the adenosine receptor A2A. In the non-canonical pathway, NAD+ substrate is converted to ADP-ribose (ADPR) via CD38, which is then converted to AMP by CD203a and then hydrolyzed by CD73 to produce adenosine [12,18].

In regard to outcomes, high ADORA2A expression (unexpectedly) correlated with longer OS from time of advanced/metastatic in our pan-cancer patients, but did not correlate significantly with the outcome (PFS or OS) after immunotherapy. These observations suggest that ADORA2A levels might be a general prognostic factor for outcomes, but not a predictive factor for an immunotherapy benefit or lack thereof. In contrast to our results, Mittal et al. demonstrated that higher ADORA2B gene expression levels correlate with shorter OS in a triple-negative breast cancer subset; however, this finding did not hold for luminal A, B, or HER2+ subsets of breast cancer [7]. The limited number of breast cancers in our study precluded examining this particular subset of malignancies as a separate cohort. Prior studies have also suggested that high CD73, an adenosine-synthesizing enzyme, is also associated with a poor prognosis in high-grade serous ovarian cancer [31]. Our study did not analyze CD73 expression, but this molecule merits further investigation in future work. Our finding that high ADORA2A correlated with a better prognosis was perhaps surprising, but prior work suggests that ADORA2A function may be more nuanced than previously assumed. For instance, Allard and colleagues [19] recently showed that ADORA2A is a suppressor of NASH-associated hepatocellular cancer and that low ADORA2A correlates with poorer survival, consistent with our results in our pan-cancer cohort; they suggest that ADORA2A may have a previously unrecognized tumor suppressor function in the liver. The fact that ADORA2A expression did not correlate with the immunotherapy outcome suggests that this expression specifically does not affect the impact of anti PD1 agents, which most of our patients received.

There are important limitations to our study. Amongst these is the fact that we assessed bulk RNA; single-cell testing is merited in the future to determine the cell of origin of ADORA2A expression. Furthermore, our work was performed in a pan-cancer cohort, perhaps pointing to the generalizability of the observations, but precluding our ability to analyze prognostic and predictive correlations for ADORA2A in individual histologies. Future studies should address larger cohorts of individual histologies. Another limitation of this study was that we did not have the tissues to perform protein analysis. Such analysis should be a subject for future investigation.

In conclusion, our study demonstrated that 20.8% of diverse cancers expressed high levels of ADORA2A transcripts, with the greatest proportions of high expressors in neuroendocrine and breast cancers and in sarcomas, and with colorectal and ovarian cancers having the lowest proportion of high expressors. However, expression patterns varied between and within tumor types, indicating the need for tumor-by-tumor immunomic testing. Most tumors expressed a complex array of immune molecules, and high ADORA2A was associated with high levels of PD-1 and VISTA checkpoints, as well as with high levels of CD38 and CD39 enzymes, the latter being critical to the adenosine pathway; these associations may be important when considering optimized combination immunotherapy regimens. Finally, our study found no specific predictive correlation between ADORA2A levels and outcomes after immune checkpoint blockade treatment. However, unexpectedly, high ADORA2A RNA levels may be a general favorable prognostic factor for better survival outcomes in patients with advanced/metastatic cancers, consistent with prior data showing that ADORA2A function is more nuanced than initially assumed, and may have a tumor suppressor effect [19] in addition to its pro-tumorigenic effects. Taken together, the current investigation indicates that individualized testing for ADORA2A levels, as well as for co-expressed immunomodulatory molecules, including specific checkpoints, may be required in order to optimize precision immunotherapy-based treatment selection, in the same way that tumor genomic sequencing is needed for selecting gene-targeted treatments as part of the precision genomics paradigm [32,33].

## 4. Materials and Methods

### 4.1. Patients

The RNA expression levels of ADORA2A in 514 solid tumor samples were analyzed as part of the clinical work-up of patients seen at the University of California Moores Cancer Center for Personalized Cancer Therapy. Only cancer types with >10 tissue samples were included in the final analysis. This was a real-world study that evaluated patients that were selected for immunomic analysis by their physicians. Tissues were evaluated at a College of American Pathologist (CAP)-accredited and Clinical Laboratory Improvement Amendments (CLIA)-licensed clinical laboratory, OmniSeq (https://www.omniseq.com/, accessed on 20 April 2024), Labcorp Oncology, Buffalo, NY, USA. This study and any investigational interventions, for which patients gave consent, were conducted in accordance with the UCSD Institutional Review Board guidelines (UCSD_PREDICT, NCT02478931: Study of Personalized Cancer Therapy to Determine Response and Toxicity).

### 4.2. Transcriptomics

Formalin-fixed, paraffin-embedded (FFPE) tissue specimens were collected, and the RNA was extracted using a truXTRAC FFPE extraction kit (Covaris, Inc., Woburn, MA, USA) as per the manufacturer’s instructions. After purification, the RNA was dissolved in 50 µL of water and the yield was measured via Quant-iT RNA HS assay (manufactured by Thermo Fisher Scientific, Waltham, MA, USA). For library preparation, the pre-defined titer of 10ng of RNA was considered acceptable for the transcriptome sequencing of a clinically validated 395-gene expression panel relating to the anticancer immune response, as previously described [34]. Following sequencing on an Ion Torrent S5XL system, RNA sequencing absolute reads were generated using the immuneResponseRNA (v5.2.0.0) plug-in of Torrent Suite Software (Thermo Fisher Scientific, Waltham, MA, USA). The RNA expression levels of ADORA2A, LAG-3, IDO1, VISTA, TIM-3, PD-1, PD-L1, PD-L2, and CTLA-4 were evaluated. RNA transcripts were standardized to internal housekeeping genes; transcript levels were then ranked on a 0 to 100 scale standardized to 735 specimens from 35 different cancer types. The transcript expression profiles were stratified by transcript abundance rank values into “Low or Moderate” (0–74) and “High” (75–100) percentile RNA expression ranks. The odds ratio for high ADORA2A expression was calculated for multiple genes and cancer histologies with >10 samples. If more than one unique sample was analyzed from the same patient on different days, then the earlier time-stamped sample was included in this analysis.

### 4.3. Tumor Mutational Burden (TMB)

For tumor mutational burden (TMB) analysis, genomic DNA was obtained from qualified FFPE tumors (>30% tumor nuclei) by means of a truXTRAC FFPE extraction kit (Covaris) with 10 ng DNA input for library preparation. DNA libraries were later created with Ion AmpliSeq targeted sequencing chemistry by employing the Comprehensive Cancer Panel, followed by enrichment and template preparation utilizing the Ion Chef system and sequencing on an Ion S5XL 540 chip (manufactured by Thermo Fisher Scientific, Waltham, MA, USA). TMB was reported as eligible mutations per qualified panel size (mutations/megabase) after the removal of synonymous variants, germline variants, indels, and single nucleotide variants with <5% variant allele fractions. High TMB was defined as ≥10 mutations/megabase (mut/MB) and TMB <10 (mut/MB) was categorized as low/moderate.

### 4.4. Data Collection and Analysis

The dataset included information on ADORA2A RNA expression; cancer histology; patient demographics; and common immune biomarkers such as microsatellite instability (MSI) status, tumor mutational burden (TMB), and programmed death-ligand 1 (PD-L1) immunohistochemistry (IHC) status. Binary logistical regression was used to calculate the odds ratio (OR) with a 95% confidence interval (CI). Descriptive studies, including cross tabs and frequency, were used for the calculation of percentages. Outcome variables, including overall survival (OS) and progression-free survival (PFS) after immune checkpoint blockade, were also curated. This database has been previously reported [35,36,37,38]. For the evaluation of OS and PFS, Kaplan–Meier analysis was used. Patients still alive (for OS) or progression-free (for PFS) at the time of data cut-off or last follow-up were censored at that time point. Data variables with a *p*-value ≤ 0.05 in univariate analysis were evaluated in the multivariate logistical regression model for independent correlations.

## Figures and Tables

**Table 1:** ADORA2A RNA expression and clinical features (N = 514 patients).

Clinical Characteristics		Univariable		Multivariable		Comment
Condition (N = Total No. of Cases)	Proportion of High ADORA2A among All Cases with (%)	Odds Ratio (95% CI)	*p*-Value	Odds Ratio (95% CI)	*p*-Value	
Gender Men (N = 204) Women (N = 310)	Men (46/204, 22.5%) Women (61/310, 19.6%)	1.19 (0.77–1.83)	0.433			
Age above 61 (N = 256) and below median of 61 years (N = 258)	Age above median 61 (60/256, 23.4%) Age below median 61 (72/258, 27.9%)	0.82 (0.53–1.25)	0.351			
^+^ PD-L1 (CPS ≥ 1%) IHC (N = 156) PD-L1 (<1%) IHC (N = 357)	^+^ PDL1 (≥1%) IHC (31/156, 19.8%) PDL1 (<1%) IHC (76/357, 21.2%)	0.92 (0.57–1.45)	0.716			One patient was missing PD-L1 IHC evaluation
High ^1^ CTLA-4 (N = 87) Low/Moderate CTLA-4 (N = 427)	High ^1^ CTLA4 (33/87, 37.9%) Low/Moderate CTLA4 (74/427, 17.3%)	2.92 (1.76–4.80)	**<0.001**	0.79 (0.37–1.64)	0.538	
High ^1^ LAG-3 (N = 116) Low/Moderate LAG-3 (N = 398)	High ^1^ LAG3 (41/116, 35.3%) Low/Moderate LAG3 (66/398, 16.5%)	2.75 (1.72–4.36)	**<0.001**	0.86 (0.42–1.70)	0.665	
High ^1^ PD-1 (N = 93) Low/Moderate PD-1 (N = 421)	High ^1^ PD-1 (41/93, 44.08%) Low/Moderate PD-1 (66/421, 15.6%)	4.24 (2.60–6.90)	**<0.001**	2.55 (1.19–5.45)	**0.015**	**High ADORA2A RNA (≥75th percentile rank) was positively associated with high PD1**
High ^1^ PD-L1 (N = 67) Low/Moderate PD-L1 (N = 447)	High ^1^ PD-L1 (25/67, 37.3%) Low/Moderate PD-L1 (82/447, 18.3%)	2.65 (1.51–4.57)	**<0.001**	0.198 (0.74–3.86)	0.198	
High ^1^ PD-L2 (N = 100) Low/Moderate PD-L2 (N = 414)	High ^1^ PD-L2 (37/100, 37.0%) Low/Moderate PD-L2 (70/414, 16.9%)	2.89 (1.78–4.66)	**<0.001**	1.07 (0.51–2.21)	0.858	
High ^1^ TIM-3 (N = 90) Low/Moderate TIM-3 (N = 424)	High ^1^ TIM3 (28/90, 31.1%) Low/Moderate TIM3 (79/424, 18.6%)	1.97 (1.17–3.26)	**0.009**	0.50 (0.23–1.05)	0.073	
High ^1^ VISTA (N = 166) Low/Moderate VISTA (N = 348)	High ^1^ VISTA (59/166, 35.5%) Low/Moderate VISTA (48/348, 13.7%)	3.45 (2.22–5.37)	**<0.001**	3.05 (1.75–5.36)	**0.001**	**High ADORA2A RNA (≥75th percentile rank) was positively associated with high VISTA**
High ^1^ CD38 (N = 79) Low/Moderate CD38 (N = 435)	High ^1^ CD38 (35/79, 44.3%) Low/Moderate CD38 (72/435, 16.5%)	4.01 (2.40–6.69)	**<0.001**	2.24 (1.07–4.63)	**0.031**	**High ADORA2A RNA (≥75th percentile rank) was positively associated with high CD38**
High ^1^ CD39 (N = 131) Low/Moderate CD39 (N = 383)	High ^1^ CD39 (54/131, 41.2%) Low/Moderate CD39 (53/383, 13.8%)	4.37 (2.78–6.89)	**<0.001**	3.54 (1.93 -6.54)	**<0.001**	**High ADORA2A RNA (≥75th percentile rank) was positively associated with high CD39**
High ^1^ IDO1 (N = 91) Low/Moderate IDO1 (N = 423)	High ^1^ IDO1 (19/91, 20.8%) Low/Moderate IDO1 (88/423, 20.8%)	1.00 (0.56–1.72)	0.987			
Microsatellite unstable (N = 15) Microsatellite stable (N = 425)	Unstable (3/15, 20.0%) Stable (85/425, 20.0%)	1.00 (0.22–3.23)	0.999			
TMB (N = 450 total samples) ^a^ TMB ≥ 10 mutations/megabase (N = 33) TMB < 10 mutations/megabase (N = 417)	TMB ≥10 mutations/megabase (8/33, 24.2%) TMB < 10 mutations/megabase (81/417, 19.4%)	1.33 (0.54–2.93)	0.505			
Neuroendocrine cancer (N = 15)	NET (11/15, 73%) Not NET (96/499, 19.2%)	11.5 (3.86–42.4)	**<0.001**	19.3 (5.53–80.4)	**<0.001**	**High ADORA2A RNA (≥75th percentile rank) was positively associated with neuroendocrine cancer**
Sarcoma (N = 24)	Sarcoma (9/24, 37.5%) Not Sarcoma (98/490, 20.0%)	2.40 (0.98–5.56)	**0.045**	3.34 (1.21–8.91)	**0.017**	**High ADORA2A RNA (≥75th percentile rank) was positively associated with sarcoma**
Breast cancer (N = 49)	Breast (19/49, 38.8%) Not Breast (88/465, 18.9%)	2.71 (1.44–5.01)	**0.002**	4.43 (2.01–9.78)	**<0.001**	**High ADORA2A RNA (≥75th percentile rank) was positively associated with breast cancer**
Pancreas cancer (N = 55)	Pancreas (13/55, 23.6%) Not Pancreas (94/459, 20.4%)	1.20 (0.60–2.27)	0.586			
Carcinoma Unknown Primary or CUP (N = 13)	CUP (3/13, 23.1%) Not CUP (104/501, 20.7%)	1.15 (0.25–3.82)	0.839			
Esophageal cancer (N = 17)	Esophageal ca (2/17, 11.8%) Not esophageal ca (105/497, 21.1%)	0.50 (0.08–1.80)	0.359			
Lung cancer (N = 20)	Lung ca (3/20, 15%) Not Lung CA (104/494, 21%)	0.66 (0.15–2.02)	0.516			
Head and neck cancer (N = 12)	Head & Neck cancer (2/12, 16.7%) Not head & neck cancer (105/502, 20.9%)	0.76 (0.12–2.92)	0.721			
Stomach cancer (N = 25)	Stomach ca (5/25, 20%) Not stomach ca (102/489, 20.8%)	0.95 (0.31–2.41)	0.918			
Liver and bile duct cancer (N = 19)	Liver ca (4/19, 21.1%) Not liver Ca (103/495, 20.8%)	1.01 (0.28–2.87)	0.979			
Uterine cancer (N = 24)	Uterine cancer (3/24, 12.5%) Not uterine cancer (104/490, 21.2%)	0.53 (0.12–1.58)	0.312			
Small intestine cancer (N = 12)	Small intestine (2/12, 16.7%) Not small intestine cancer (105/502, 20.9%)	0.76 (0.12–2.92)	0.721			
Colorectal cancer (N = 140)	Colorectal (15/140, 10.7%) Not colorectal (92/374, 24.5%)	0.37 (0.20–0.64)	**0.001**	0.49 (0.24–0.96)	**0.044**	**High ADORA2A RNA (≥75th percentile rank) was negatively associated with colorectal cancer**
Ovarian cancer (N = 43)	Ovarian cancer (2/43, 4.7%) Not ovarian cancer (105/471, 22.2%)	0.17 (0.03–0.57)	**0.005**	0.17(0.02–0.67)	**0.028**	**High ADORA2A RNA (≥75th percentile rank) was negatively associated with ovarian cancer**

Notes: ^1^ High refers to RNA/transcript expression level ≥ 75th percentile of controls. Low/moderate refers to transcript/RNA expression level < 75th percentile of controls. ^+^, for PDL1 IHC (total N = 513, CPS score ≥ 1), there was one value with unknown CPS score that was marked as missing. ^a^ Total number of patients were less in some categories (e.g., TMB) because data were only available for 450 cases. Multivariate analysis was performed only among patients with variables with ***p* value ≤ 0.05** on univariate analysis. **Abbreviations:** CPS: combined positive score; CTLA4: cytotoxic T-lymphocyte-associated protein 4; IDO1: indoleamine 2, 3-dioxygenase 1; LAG3: lymphocyte activation gene 3; PDL: programmed cell death ligand; PD: programmed cell death protein; TMB: tumor mutational burden; TIM3: T cell immunoglobulin and mucin domain 3; VISTA: V-domain Ig suppressor of T cell activation.

## Data Availability

The raw data presented in this study is available on request from the corresponding author.

## Supplementary Materials

The following supporting information can be downloaded at: https://www.mdpi.com/article/10.3390/ijms25094742/s1.

## Abbreviations

Castrate-resistant prostate cancer, CRPC; colorectal cancer, CRC; head and neck squamous cell carcinoma, HNSCC; non-small cell lung cancer, NSCLC; renal cell carcinoma, RCC; triple-negative breast cancer, TNBC.
