# Supplementary material for: Clinical and Biologic Correlates of ADORA2A Transcriptomic Expression in Cancer
Source: Int J Mol Sci. 2024 Apr 26;25(9):4742. doi: 10.3390/ijms25094742 (PMC11083822; doi:10.3390/ijms25094742)
Supplement: Supplementary file 1 [file ijms-25-04742-s001.zip › ijms-2954182-supplementary.pdf]

Supplementary Table S1: Patient Demographics

Variable	N = 514[1]
Age (median (range))	61 (24, 93)
Sex	
Female	310 (60%)
Male	204 (40%)
PD-L1 immunohistochemistry >=1	156 (30%)
(Missing)	1
Microsatellite instability high	15 (3.4%)
(Missing)	74
Tumor mutational burden $\geq$10 mutations/mb	33 (7.3%)
(Missing)	64
High ADORA2A expression	
High ($\geq$75th percentile rank)	107 (20.8%)
Low	407 (79.2%)
**Cancer Types**	
Adrenal Gland Cancer	3 (0.6%)

Variable	N = 514[1]
Basal Cell Carcinoma of the Skin	1 (0.2%)
Bladder Cancer	4 (0.8%)
Brain and Nervous System Cancer	3 (0.6%)
Breast Cancer	49 (9.5%)
Cervical Cancer	5 (1.0%)
Colorectal Cancer	140 (27%)
Esophageal Cancer	17 (3.3%)
Gallbladder/Extrahepatic Bile Duct Cancers	4 (0.8%)
Head and Neck Cancer	12 (2.3%)
Kidney and Renal Pelvis Cancer	3 (0.6%)
Lipomatous Neoplasms	2 (0.4%)
Liver and Bile Duct Cancer	19 (3.7%)
Lung Cancer	20 (3.9%)
Melanoma	6 (1.2%)
Mesothelioma	2 (0.4%)
Neuroendocrine Tumors	15 (2.9%)

Variable	N = 514[1]
Ocular melanoma	1 (0.2%)
Ovarian Cancer	43 (8.4%)
Pancreatic Cancer	55 (11%)
Primary peritoneal carcinoma	1 (0.2%)
Prostate Cancer	4 (0.8%)
Sarcoma	24 (4.7%)
Small Intestine Cancer	12 (2.3%)
Squamous Cell Carcinoma of the Skin	3 (0.6%)
Stomach Cancer	25 (4.9%)
Thymic Cancer	1 (0.2%)
Thyroid Cancer	3 (0.6%)
Carcinoma of unknown primary (CUP)	13 (2.5%)
Uterine Cancer	24 (4.7%)

Supplementary Table S2. Examples of completed or ongoing trials of adenosine receptor antagonists in oncology

Drug	Target	Diseases	Company	Number of clinical trials registered in NCT (clinicaltrials.gov)	**Comment/Results**
NIR178 in combination with PDR001<br><br>Phase II<br><br>Active but not recruiting.	NIR178 is an A2A adenosine receptor antagonist.<br><br>PDR001 is an anti PD1	NSCLC<br>RCC<br>Pancreatic Cancer<br>Urothelial Cancer<br>HNSCC<br>Diffused Large B Cell Lymphoma<br>Microsatellite Stable Colon Cancer<br>TNBC<br>Melanoma<br>Metastatic CRPC	Novartis	NCT03207867	
Etrumadenant (AB928) in combination with mFOLFOX<br><br>Phase I/Ib completed.	Etrumadenant is an **A2A** and A2B adenosine receptor antagonist.	Advanced stage Gastroesophageal cancer Colorectal cancer.	Arcus Biosciences, Inc.	NCT03720678	None yet
PBF-509 +PDR001<br><br>Phase I completed.	PBF-509 (A2A adenosine receptor antagonist)	NSCLC	Novartis	NCT02403193	
Drug: Ciforadenant (arm 1) Ciforadenant +Atezolizumab (arm 2)	Ciforadenant, an oral small molecule targeting the-A2A adenosine receptor.	RCC<br>CRPC	Genentech	NCT02655822	For cifrodenant monotherapy arm high-grade arthralgia, peripheral edema and anemia were

Phase I completed					observed in 3% cases.<br>For combination arm grade ¾ abdominal, pain, high AST, hypophosphatemia and arthralgia was observed in 2.9% cases.<br><br>Ciforadenant plus atezolizumab showed a partial response of 11%
SRF617 in combination with etrumadenant and zimberelimab Phase II Recruiting	Etrumadenant is an A2aR and A2bR antagonist.<br>SRF 617 is CD39 inhibitor.<br><br>Zimberelimab is a fully human anti-PD-1 monoclonal antibody.	Metastatic CRPC	Arcus Biosciences, Inc.	NCT05177770	
Zimberelimab + Domvanalimab + Etrumadenant<br><br>Phase 2 Recruiting	Etrumadenant is an **A2A** and A2B adenosine receptor antagonist.<br>Zimberelimab is an anti PD1 antibody, Domvanalimab is anti TIGIT antibody.	NSCLC	Arcus Biosciences	NCT04262856	
DFF332 + +PDR001 (Spartalizumab) +NIR178 (Taminadenant)	DFF332(Hif2alpha inhibitor) + PDR001 is anti PD1 inhibitor + NIR178 which is an adenosine A2A adenosine receptor antagonist.	RCC	Novartis	NCT04895748	
Etrumedanant + Zimberelimab	Etrumedanant is an A2A adenosine receptor antagonist.	NSCLC, HNSCC, CRC, Melanoma, Bladder Cancer,	Arcus Bio	NCT03629756	

Phase I completed	Zimberelimab is an anti PD1 antibody	Ovarian Cancer, Endometrial Cancer, RCC, CRPC			
Etrumadenant IPI- 549 Pegylated Liposomal Doxorubicin (PLD) Nanoparticle albumin-bound paclitaxel	Etrumedanant is an A2A adenosine receptor antagonist.<br>IPI-549 is PI3kinase gamma inihibitor	TNBC<br>Ovarian Cancer	Arcus Bio	NCT03719326	
Preladenant, pembrolizumab Phase I	Preladenant is A2A adenosine receptor antagonist.	Solid tumors	Merck Sharp & Dohme Corp.	NCT03099161	
AZD4635	A2A adenosine receptor antagonist.	Solid tumors	Astrazeneca	NCT03980821	
Regorafenib, atezolizumab, imprime PGG, bevacizumab, isatuximab, selicrelumab, idasanutlin, AB928	AB928 is a small-molecule A2A/A2B adenosine receptor antagonist	CRC	Hoffmann–La Roche	NCT03555149	
PBF1129. Nivolumab	A2B Receptor Inhibitor	NSCLC	Palobiofarma	NCT05234307	
Inupadenant	A2A Receptor Inhibitor	Solid tumors	iTeos Therapeutics	NCT05060432	

Foot note: Data search as of June17th, 2023 (Pubmed and Clinicaltrials.gov).

Abbreviations: Castrate-resistant prostate cancer (CRPC); Colorectal cancer (CRC); Head and neck squamous cell carcinoma (HNSCC); Non-small cell lung cancer (NSCLC); Renal cell carcinoma (RCC); Triple-negative breast cancer (TNBC).
